# Red Blood Cell Deformation and Progressive Anemia Following Therapeutic Intervention in Patients With Adult T-Cell Leukemia/Lymphoma

**DOI:** 10.7759/cureus.34641

**Published:** 2023-02-05

**Authors:** Kosuke Obama, Seiitiro Nakabeppu, Hirosaka Inoue

**Affiliations:** 1 Department of Hematology, Imakiire General Hospital, Kagosima, JPN

**Keywords:** anemia, red cell distribution width (rdw), red blood cell deformation, tumor lysis, red blood cell, adult t-cell leukemia/lymphoma

## Abstract

Objectives: During therapeutic intervention for adult T-cell leukemia-lymphoma (ATLL), transient red blood cell (RBC) deformations and rapid anemia progression are often observed. These RBC responses are characteristically observed during the treatment of ATLL, and we examined the details and significance of these RBC responses.

Methods: Seventeen patients with ATLL were enrolled. Peripheral blood smears and laboratory findings were collected during the first two weeks after treatment intervention. We examined the transition of erythrocyte morphology and the factors associated with the induction of anemia.

Results: RBC abnormalities (i.e., elliptocytes, anisocytosis, and schistocytes) rapidly progressed following therapeutic intervention in five of the six cases for whom evaluable consecutive blood smears were available, with significant improvement evident after two weeks. Changes in RBC morphology were significantly associated with the red cell distribution width (RDW). Laboratory findings from all 17 patients showed various levels of anemia progression. A transient increase in RDW values was observed in 11 cases after therapeutic intervention. The degree of progressive anemia during the two-week period was significantly correlated with increased lactate dehydrogenase and soluble interleukin-2 receptor levels and an increase in RDW (P <0.01).

Conclusions: In cases of ATLL, transient progression of RBC morphological abnormalities and RDW value were observed early after therapeutic intervention. These RBC responses may be associated with tumor and tissue destruction. RBC morphology or RDW values may provide important information about the tumor dynamics and general condition of the patients.

## Introduction

Adult T-cell leukemia/lymphoma (ATLL) is a hematological malignancy with poor prognosis associated with a human T-lymphotropic virus infection [1]. Malignant cells have the characteristics of peripheral T-cell leukemia/lymphoma [2,3] and generally have little hematopoietic effects. Even though highly progressive anemia is often observed following treatment intervention, it is assumed that there are a certain number of cases in which ATLL cells have hematopoietic inhibition. The progression of anemia is rapid, and we observe a high degree of red blood cell (RBC) morphological variability after the therapeutic intervention; therefore, complex factors related to tumor disruption are assumed to be involved in the mechanism. These RBC responses are not observed during therapeutic intervention for other lymphoid malignancies. We performed a detailed analysis of anemia progression associated with therapeutic intervention in patients with ATLL.

## Materials and methods

We analyzed patients with acute or lymphoma-type ATLL who were diagnosed at our hospital between 2014 and 2020 and underwent initial treatment with a CHOP (doxorubicin, cyclophosphamide, vincristine, prednisone)-based chemotherapy regimen. Patients who received mogamulizumab in combination with CHOP therapy were also included in this analysis. Patients with any of the following histories were excluded: 1) receiving any anti-cancer drugs, immunosuppressants, or radiation therapy within the last five years; and 2) having any serious infectious diseases within the past 12 months. We also excluded patients with iron or vitamin deficiencies at the time of initial diagnosis. To minimize the effect of anticancer drugs on hematopoiesis and RBC morphology, peripheral blood smears and laboratory data were collected within two weeks of treatment intervention. Based on past clinical observations, these two weeks was the time when the actual major changes in RBC morphology have been observed. Blood smears were collected exclusively from the patients with acute-type ATLL who were available for the appraisable peripheral blood smears and were evaluated by a physician and authorized laboratory technologists. Data on the peripheral blood cell count, mean corpuscular volume (MCV), red cell distribution width (RDW), hemoglobin (Hb), lactate dehydrogenase (LDH), and soluble interleukin-2 receptor (sIL-2R) were collected, and the trends of these data over time were examined. To examine the factors influencing the progression of anemia, the association between the degree of progression of anemia and LDH, sIL2R, and RDW was statistically analyzed. Statistical analyses were conducted using the Shapiro test and cor test functions of R version 4.2.0.

## Results

**Figure 1:**
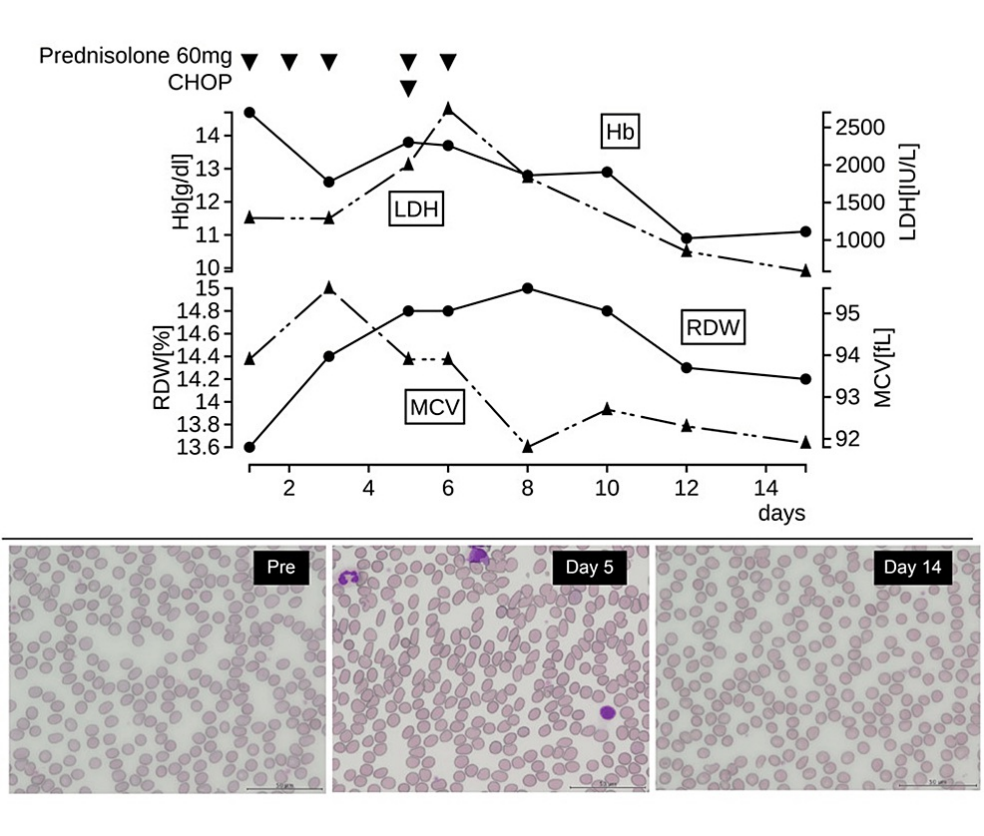
Clinical course of a representative case of acute-type adult T-cell leukemia/lymphoma Peripheral blood smears show mild red blood cell deformation at diagnosis, followed by morphological deterioration after therapeutic intervention and restoration of the shape of red blood cells on day 14.

Seventeen patients with ATLL (11 with acute-type ATLL and six with lymphoma-type ATLL) were enrolled. None of these cases had exclusion criteria. Since our hospital does not support transplantation, the enrolled patients were predominantly elderly people (median age, 76 years; range, 67-90 years). First, we examined changes in RBC morphology and laboratory findings in six acute-type cases for which we were able to evaluate peripheral blood smears over time. The clinical course of a representative case of acute-type ATLL is shown in Figure 1.

The patient was a 70-year-old man with acute-type ATLL. Laboratory examination showed a white blood cell count of 18.66 × 109/L with 40% abnormal lymphocytes. Mild RBC morphological abnormalities were evident at the time of diagnosis, and these morphological abnormalities (i.e., elliptocytes, anisocytosis, and schistocytes) rapidly progressed following therapeutic intervention. Improvements in RBC morphological abnormalities were obvious after two weeks, during which the Hb level decreased by 3.8 g/dL. RBC morphological abnormalities were linked to LDH movement, which was, in turn, associated with tumor collapse. MCV showed a rapid decline after treatment intervention, indicating a shift towards its normocytic. RDW also showed a transient increase in conjunction with changes in RBC morphology. Five of the six cases showed transient RBC morphological abnormalities over the time course of therapeutic intervention. Similar to the case described above, these fluctuations were associated with the RDW. Although strict quantitative evaluation of changes in RBC morphology is difficult, we concluded that RDW could be an evaluable surrogate marker of morphological change.

**Figure 2:**
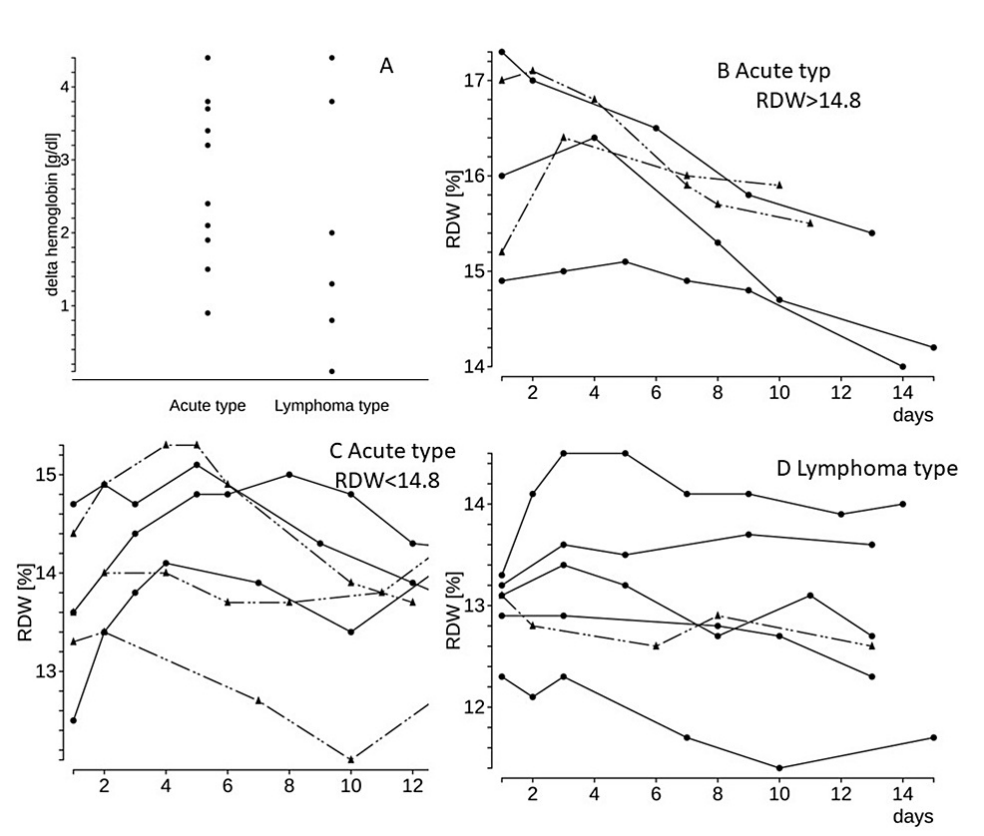
Progression of anemia and changes in red cell distribution width values after therapeutic intervention for adult T-cell leukemia/lymphoma (A) Progression of anemia during the first two weeks of treatment. (B, C, D) Changes in the red cell distribution width (RDW) value of the patients with acute and lymphoma type of adult T-cell leukemia/lymphoma. The RDW tended to be higher at diagnosis in acute types. Eleven of 17 cases showed the temporal surge of the RDW throughout the initial treatment.

Next, the detailed laboratory findings of all 17 cases were examined. The decrease in Hb levels over the two-week course of treatment intervention is shown in Figure 2A.

Highly progressive anemia was slightly more frequent among patients with acute-type ATLL (0.1-4.4 mg/dL, median 3.2 mg/dL). However, there were also cases of highly progressive anemia in patients with lymphoma-type ATLL (0.1-4.4 mg/dL, median 1.7 mg/dL). To estimate the transition of RBC morphological abnormalities throughout the treatment, changes in the trend of RDW as a simple and quantitative surrogate marker are shown in Figure 2. To improve the visibility of RDW trends, acute-type ATLL was classified into two groups based on RDW values at the time of diagnosis. As a result, short-term fluctuations in the RDW were observed throughout the treatment in almost all cases (Figures 2B, 2C, 2D). In acute-type ATLL, there were many cases with a high RDW at the time of diagnosis, which quickly decreased after therapeutic intervention. Two patients with RDW >17 % had poor PS, and both were transitioned to palliative care early after a short period of treatment. Of the 11 cases of acute-type ATLL, eight were accompanied by a transient increase in RDW of at least 0.2%. In the lymphoma type, the trend was divided, but three cases showed transient elevation of RDW as in the acute type. These three cases demonstrated soluble IL2R >100,000 IU/L and delta Hb >2 g/dL, suggesting a strong association with tumor volume and induction of anemia.

**Figure 3:**
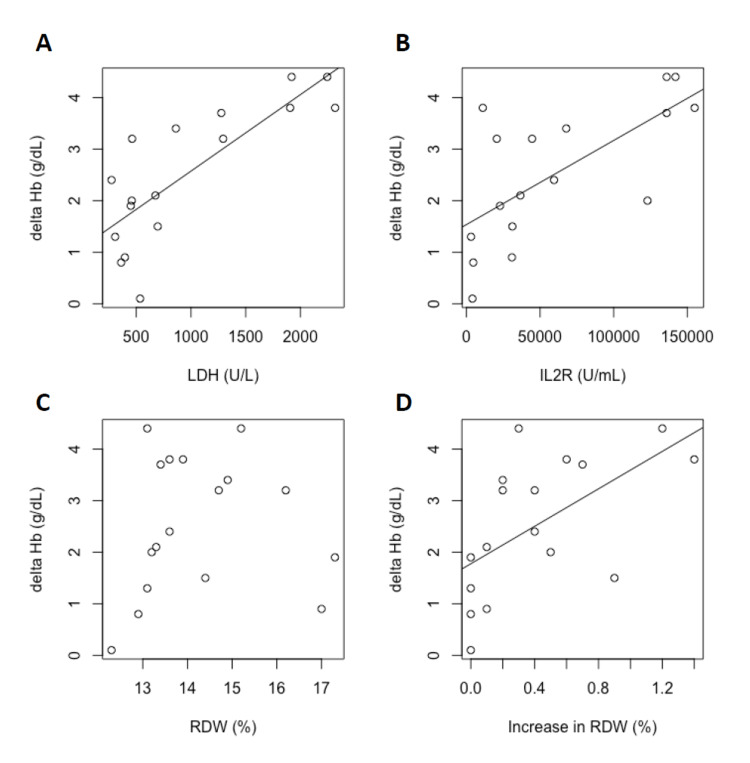
Correlation and regression analyses to infer causal factors of progressive anemia observed during two weeks after the initial treatment. Equations are the linear regression models. (A, B) The decrease in hemoglobin level was significantly correlated with the increase in lactate dehydrogenase (LDH) and soluble interleukin-2 receptor (sIL-2R) levels. Spearman’s correlation: LDH, rho = 0.78, P < 0.001; sIL-2R, rho = 0.71, P = 0.0012. (C, D) The red cell distribution width (RDW) value at diagnosis was not correlated with the progression of anemia (Pearson’s correlation, P = 0.76), but an increase in RDW showed a significant correlation with the progression of anemia (Spearman’s correlation, rho = 0.69, P = 0.002).

The degree of anemia progression during the two weeks of treatment was significantly correlated with an increase in LDH and sIL-2R levels (Figures 3A, 3B).

Although the RDW at the time of diagnosis was not significantly correlated with the progression of anemia, the range of increase in RDW (i.e., the difference between the RDW value at the time of diagnosis and the RDW peak value) showed a significant association with anemia progression (Figures 3C, 3D).

## Discussion

In the present study, we focused on the RBC dynamics throughout the therapeutic intervention in patients with ATLL. Based on the examination of cases for which peripheral blood smears were available, RDW was considered a simple and quantitative surrogate marker of RBC morphological changes. As a result, although RBCs are highly morphologically stable due to their unique membrane structure [4], progressive RBC deformations and large fluctuations in RDW values were observed throughout the treatment. Additionally, the significant correlations between increased LDH increased sIL-2R, and decreased Hb levels suggested that the degree of anemia progression was associated with a higher tumor cell volume or higher tumor activity. We also showed that an elevation in the RDW after intervention significantly correlated to the progression of anemia. Although the influence of direct tumor cell infiltration into the bone marrow cannot be ruled out, tumor lysis may have a potential effect on enhancing RBC deformation and promoting the progression of anemia attributable to cells being immediately captured by the reticuloendothelial system. The fact that similar results were also observed in the lymphoma-type group supports this inference. In the acute-type group, many patients already had RBC morphological abnormalities at the time of diagnosis, which may reflect chronic tumor disruption associated with tumor immunity or fragility in the process leading to diagnosis. In addition, the patients with particularly severe RBC morphologic abnormalities at the time of diagnosis (RDW >17%) had a poor PS, suggesting that, in addition to tumor collapse, the patient's progressive tissue destruction might influence RBC deformations.

Several mechanisms may be postulated for this RBC response. A recent study regarding toll-like receptor 9 (TLR9) expression on RBC membranes is of great interest in explaining the potential mechanisms underlying our finding [5]. TLR9-expressing RBCs recognize specific nucleotide sequences originating in microbiota, which ultimately promotes RBC clearance in the reticuloendothelial system through RBC deformation and deregulation of CD47 expression. Considering that the specific nucleotide sequence also exists in human DNA (mainly in the mitochondria), we can assume that RBC deformation and anemia progression are both induced by the escalated concentration of the cell-free DNA resulting from tumor lysis and tissue damage. The RBCs of patients with ATLL are constantly exposed to the cell-free DNA due to tumor-cell vulnerabilities or the effect of anti-tumor immunity by the time of initial diagnosis, which possibly promotes abnormalities in RBC shape and phenotypic traits. Such dynamic RBC changes cannot be confirmed in other diseases (e.g., B-cell lymphoma); however, it is assumed that RBCs are also affected by tumor and tissue destruction in various diseases.

In recent years, RDW has been identified as a poor prognostic factor for cardiovascular disease [6,7], hematological malignancies [8-11], and other various malignant diseases [12-15], but the reasons for this remain unclear. Considering these results, it is possible that changes in RBC morphology and increases in RDW may be observed as a result of the destruction of tumor cells and sometimes normal tissue in patients with various diseases. In other words, RBC morphology or RDW values may provide important information about the tumor dynamics and general condition of the patients. It is conceivable that there are still unknowns in the role of RBCs that need to be elucidated.

## Conclusions

In cases of ATLL, transient progression of RBC morphological abnormalities and an increase in RDW value were observed early after therapeutic intervention. In addition, anemia progression was often identified in both acute and lymphoma types, and the degree of progressive anemia during the two weeks was significantly correlated with increased lactate dehydrogenase and soluble interleukin-2 receptor levels and an increase in RDW (P <0.01). These RBC responses may be associated with tumor and tissue destruction. RBC morphology or RDW values may provide important information about the tumor dynamics and general condition of the patients.
